# Genomes of the Caribbean reef-building corals *Colpophyllia natans*, *Dendrogyra cylindrus*, and *Siderastrea siderea*

**DOI:** 10.1093/g3journal/jkaf020

**Published:** 2025-02-01

**Authors:** Nicolas S Locatelli, Iliana B Baums

**Affiliations:** Department of Biology, The Pennsylvania State University, University Park, PA 16802, USA; Department of Biology, The Pennsylvania State University, University Park, PA 16802, USA; Helmholtz Institute for Functional Marine Biodiversity at the University of Oldenburg (HIFMB), Carl von Ossietzky Universität Oldenburg, Im Technologie Park 5, Oldenburg 26129, Germany; Alfred Wegener Institute, Helmholtz-Centre for Polar and Marine Research (AWI), Am Handelshafen 12, Bremerhaven 27570, Germany; Institute for Chemistry and Biology of the Marine Environment (ICBM), School of Mathematics and Science, Carl von Ossietzky Universität Oldenburg, Ammerländer Heerstraße 114-118, Oldenburg 26129, Germany

**Keywords:** genome, coral, reef, gene family expansion, duplication, orthogroups, *Siderastrea siderea*, *Dendrogyra cylindrus*, *Colpophyllia natans*

## Abstract

Coral populations worldwide are declining rapidly due to elevated ocean temperatures and other human impacts. The Caribbean harbors a high number of threatened, endangered, and critically endangered coral species compared with reefs of the larger Indo-Pacific. The reef corals of the Caribbean are also long diverged from their Pacific counterparts and may have evolved different survival strategies. Most genomic resources have been developed for Pacific coral species which may impede our ability to study the changes in genetic composition of Caribbean reef communities in response to global change. To help fill the gap in genomic resources, we used PacBio HiFi sequencing to generate the first genome assemblies for 3 Caribbean reef-building corals, *Colpophyllia natans*, *Dendrogyra cylindrus*, and *Siderastrea siderea.* We also explore the genomic novelties that shape scleractinian genomes. Notably, we find abundant gene duplications of all classes (e.g. tandem and segmental), especially in *S. siderea.* This species has one of the largest genomes of any scleractinian coral (822 Mb) which seems to be driven by repetitive content and gene family expansion and diversification. As the genome size of *S. siderea* was double the size expected of stony corals, we also evaluated the possibility of an ancient whole-genome duplication using Ks tests and found no evidence of such an event in the species. By presenting these genome assemblies, we hope to develop a better understanding of coral evolution as a whole and to enable researchers to further investigate the population genetics and diversity of these 3 species.

## Introduction

Genomic resources are increasingly available for Pacific reef-building corals (e.g. Fuller *et al.* 2020; Stephens *et al.* 2022), yet most Caribbean coral species still lack them despite genetic management of their populations becoming necessary (Baums *et al*. 2022). Caribbean reefs represent ecosystems long diverged from Pacific counterparts. During the mid-Miocene, the Mediterranean closed off at both ends and the eastern connection of the Caribbean with the Indo-Pacific basin was severed (Wallace and Rosen 2006). The Isthmus of Panama to the west of the Caribbean remained open until roughly 3 million years ago, after which ocean circulation drastically changed and Caribbean reefs were isolated from Pacific reefs (Burton *et al*. 1997; O’Dea *et al*. 2016).

Cnidarians diverged early in metazoan evolution roughly 700 MYA (Park *et al*. 2012), and the 3 species discussed here represent the 2 major scleractinian lineages, complex (*Siderastrea siderea*) and robust (*Colpophyllia natans* and *Dendrogyra cylindrus*). *D. cylindrus* is a rare Caribbean coral (Hunter and Jones 1996) that has declined sharply in the past 2 decades due anthropogenic stressors and a highly infectious disease called stony coral tissue loss disease (Brandt *et al.* 2021). *D. cylindrus* is extinct in the wild in Florida and is considered critically endangered (Neely *et al*. 2021; Cavada-Blanco *et al*. 2022). *S. siderea* and *C. natans* were common reef-building corals that have also experienced significant declines in response to disease and anthropogenic impacts*. S. siderea* is now listed as critically endangered (Rodriguez-Martinez *et al*. 2022) and under threat due to acidification, ocean warming (Horvath *et al*. 2016), and stony coral tissue loss disease (Brandt *et al*. 2021). *C. natans* is also in decline due to stony coral tissue loss disease (Vermeij and Goergen 2022; Williamson *et al*. 2022). Despite their ecological and evolutionary importance, genomic resources are not yet available for these species.

Coral genomes are variable in size (e.g. Stephens *et al.* 2022), but have highly conserved gene order (Ying *et al*. 2018; Locatelli *et al*. 2024). Anthozoan genomes contain between 13.57 and 52.2% repetitive content (e.g. Shinzato *et al.* 2011; Bongaerts *et al.* 2021) and harbor DNA and retrotransposons that are still active (Chapman *et al*. 2010; Huang *et al*. 2012), which can result in gene duplication and movement of genes to disparate regions of the genome. Accumulation of somatic mutations in long-lived coral colonies represents another mechanism by which coral genomes gain heterozygosity (Devlin-Durante *et al*. 2016; López and Palumbi 2020) and some of these mutations can be passed on to their sexually produced offspring (Vasquez Kuntz *et al*. 2022). Development of genomic resources allows for further study of these complex evolutionary mechanisms in metazoans as a whole (Reusch *et al*. 2021).

To help bridge the gap in genomic resources for Caribbean corals, we present novel PacBio HiFi-derived assemblies for *C. natans*, *D. cylindrus*, and *S. siderea.* With these references, we hope to foster an understanding of how corals will respond to environmental change (Bove *et al*. 2022) and population decline (Cramer *et al*. 2020) and how the response of Caribbean corals may differ from Indo-Pacific species.

## Methods

### Tissue sampling

Tissue of *C. natans* ([latitude 12.1095 decimal degrees, longitude −68.95497 decimal degrees], database ID 22254) was collected from the Water Factory reef in Curaçao on 2022 August 6 using a hammer and chisel. *D. cylindrus* ([12.0837, −68.89447], database ID 22255) and *S. siderea* ([12.0839, −68.8944], database ID 22256) were collected from the Sea Aquarium reef in Curaçao on 2022 August 12 and 13 using a hammer and chisel. All collections were made under Curaçao Governmental Permit 2012/48584. All fragments were ca. 12 cm^2^ in size and were kept alive in coolers filled with seawater during transit prior to being preserved in DNA/RNA Shield (Zymo Research, CA, USA). Samples were stored at −20°C or at −80°C until extraction.

### Nucleic acid extraction and sequencing

For all species, DNA was extracted from tissue preserved in DNA/RNA Shield (Zymo Research) using the Qiagen (MD, USA) MagAttract HMW DNA kit, following manufacturer’s protocols. Following initial extraction, DNA was further purified using a 0.9X AMPure XP (Beckman Coulter, CA, USA) bead cleanup. Purified DNA was then size selected using a Pacific Biosciences (formerly Circulomics) SRE size selection kit. The SRE standard kit selects for DNA predominantly >25 kb and a near total depletion of fragments <10 kb. Barcoded templates were generated and sequenced by the Huck Institutes of the Life Sciences Genomics Core Facility at Penn State University using a Pacific Biosciences (Menlo Park, CA, USA) Sequel IIe across a total of 3 SMRTcells (further described below).

As RNAseq data were not available for *D. cylindrus* or any close relatives for the purposes of gene prediction, RNA was extracted from the same DNA/RNA Shield (Zymo Research) preserved samples as described above using a TriZol and a Qiagen RNeasy Mini Kit (as in https://openwetware.org/wiki/Haynes:TRIzol_RNeasy). Compared with the RNA sequence data obtained from NCBI SRA for *C. natans* and *S. siderea* (described below in “Gene prediction and functional annotation”), the RNA sample for *D. cylindrus* was of an untreated colony growing in the wild rather than experimental samples exposed to heat and disease stress. From the extracted total RNA, libraries were prepared and sequenced by the Oklahoma Medical Research Foundation Clinical Genomics Center using the NEBNext Poly(A) mRNA Magnetic Isolation Module (New England BioLabs Inc., MA, USA), Swift Rapid RNA Library Kit (Swift Biosciences, MI, USA), and 150 M read pairs of 2 × 150 bp chemistry on an Illumina (San Diego, CA, USA) NovaSeq 6000 machine.

### Genome assembly

A PacBio library was generated by pooling the barcoded templates for each of the 3 species in equal proportions and was initially sequenced on 2 SMRTcells. Prior to genome assembly, k-mer (31-mer) counting was performed on PacBio HiFi data for each species using Jellyfish v2.2.10 (Marçais and Kingsford 2011) for the purpose of haploid genome size estimation. K-mer frequency-based genome-wide heterozygosity and genome size were estimated from 31-mer histograms using GenomeScope2 (Ranallo-Benavidez *et al*. 2020). With the data from these 2 initial SMRTcells, a preliminary assembly was performed using hifiasm_meta v0.2 (Feng *et al*. 2022) to assess assembly size and to determine whether the pool balance needed to be adjusted for the third and final SMRTcell run.

Because the preliminary assembly and genome size estimate from GenomeScope2 of *S. siderea* was larger than the remaining 2 species, the final SMRTcell was run with a pool balance of 25:25:50 *Colpophyllia*/*Dendrogyra*/*Siderastrea* to provide additional coverage of the larger *Siderastrea* genome. Prior to all stages of data delivery, the sequencing facility used PacBio lima to demultiplex and remove adapters and unbarcoded sequences. Across all SMRTcells, total sequence yield was 26 Gb across 2.8 M reads in *C. natans*, 25 Gb across 2.7 M reads in *D. cylindrus*, and 32 Gb across 3.4 M reads in *S. siderea*. Further breakdown of PacBio yield and read lengths per species per sequencing run are given in Supplementary Table 1. Utilizing all data, a new set of primary assemblies was generated using hifiasm_meta.

### Assembly decontamination, haplotig purging, and repeat annotation

HiFi reads were then mapped to the assembly using minimap2 v2.24 (Li 2018), and BAM files were sorted using samtools v0.1.19 (Danecek *et al*. 2021). Using blastn v2.14.0 (Camacho *et al*. 2009), assemblies were searched against a custom database comprised of NCBI's ref_euk_rep_genomes, ref_prok_rep_genomes, ref_viroids_rep_genomes, and ref_viruses_rep_genomes databases combined with dinoflagellate and *Chlorella* genomes (Shoguchi *et al*. 2013, 2018, 2021; Hamada *et al*. 2018; Beedessee *et al*. 2020). All NCBI RefSeq databases were downloaded on 2023 March 28. Using the mapping and blastn hits files, blobtools v1.1.1 (Laetsch and Blaxter 2017) was used to identify and isolate noncnidarian contigs. To better identify symbionts within the metagenome assemblies, blastn (Camacho *et al*. 2009) was used to query putative Symbiodiniaceae contigs against a curated nuclear ribosomal Internal Transcribed Spacer-2 (ITS2) database (Hume *et al*. 2019). With all noncnidarian contigs excluded, a repeat database was modeled using RepeatModeler2 v2.0.2a (Flynn *et al*. 2020). Purge_dups v1.2.6 (Guan *et al*. 2020) was utilized to identify and remove any remaining putative haplotigs in the respective assemblies. Following haplotig purging, repeats were soft-masked using a filtered repeat library in RepeatMasker4 v4.1.2.p1 (Smit*et al*.), following recommendations from the Blaxter Lab (https://blaxter-lab-documentation.readthedocs.io/en/latest/filter-repeatmodeler-library.html). Protein references from *Orbicella faveolata* (Prada *et al*. 2016) and *Fungia* sp. (Ying *et al*. 2018) were used to filter repeat libraries for the 2 robust species (*C. natans* and *D. cylindrus*). Protein references from *Acropora millepora* (Fuller *et al*. 2020), *Montipora capitata* (Stephens *et al*. 2022), and *Galaxea fascicularis* (Ying *et al*. 2018) were used to filter repeat libraries for *S. siderea*.

### Gene prediction and functional annotation

Prior to gene prediction, the hifiasm_meta assemblies were scanned for mitochondrial contamination using MitoFinder v1.4.1 (Allio *et al*. 2020) and contigs of mitochondrial origin were removed from the assemblies. Nuclear assemblies were annotated using RNAseq data in funannotate v1.8.13 (Palmer and Stajich 2020). *C. natans* and *S. siderea* were annotated using all RNAseq data available on NCBI SRA for the respective species at the time of assembly (see Supplementary Table 2). As no RNAseq data are publicly available for *D. cylindrus* or its close relatives, RNA was extracted as previously described and included within the funannotate annotation process. All RNAseq data were adapter- and quality-trimmed using TrimGalore v0.6.7 (Krueger *et al*. 2021).

Briefly, funannotate train was run for all assemblies with a *–max_intronlen* of 100,000. Funannotate train is a wrapper that utilizes Trinity (Grabherr *et al*. 2011) and PASA (Haas *et al*. 2008) for transcript assembly. Upon completion of training, funannotate predict was run to generate initial gene predictions using the arguments --*repeats2evm*, *--organism other*, and *--max_intronlen 100000*. Funannotate predict is a wrapper that runs AUGUSTUS (Stanke *et al*. 2006) and GeneMark (Brůna *et al*. 2020) for gene prediction and EvidenceModeler (Haas *et al*. 2008) to combine gene models. Funannotate update was run to update annotations to be in compliance with NCBI formatting. For problematic gene models, funannotate fix was run to drop problematic IDs from the annotations. Finally, functional annotation was performed using funannotate annotate which annotates proteins using PFAM (Bateman *et al*. 2004), InterPro (Hunter *et al*. 2009), EggNog (Huerta-Cepas *et al*. 2019), UniProtKB (Boutet *et al*. 2016), MEROPS (Rawlings *et al*. 2010), CAZyme (Huang *et al*. 2018), and Gene Ontology (GO) (Harris *et al*. 2004). For all genes not functionally annotated with GO terms by funannotate, a single network of ProteInfer (Sanderson *et al*. 2023) was used to infer functional attributes of genes using pretrained models.

To assess the quality of genome assemblies and annotations, BUSCO v5.8.0 (Manni *et al*. 2021) was run with the metazoa_odb10 lineage dataset. BUSCO was run in genome mode on the full genome assembly and in protein mode on the predicted proteins dataset output by funannotate.

### Mitochondrial genome assembly

To assemble mitochondrial genomes for each sample, MitoHiFi v2.2 (Gabriel *et al*. 2023) was used on all available HiFi data for each species. For *S. siderea*, *C. natans*, and *D. cylindrus*, accessions NC_008167.1, NC_008162.1, and DQ643832.1 (whole mitogenomes for *Siderastrea radians*, *C. natans*, and *Astrangia poculata*) were used as seed sequences for mitochondrial assembly, respectively. For all assemblies, the arguments -a animal and -o 5 were used to indicate that the organism type was an animal and the organism genetic code was invertebrate.

### Duplication and orthogroup analysis

To assess the origin of gene duplications, whole-genome duplication pipeline and orthogroup analyses were used. The wgd pipeline v1.1 (Zwaenepoel and Van De Peer 2019) was used to investigate duplication and divergence at the whole paranome and anchor-pair levels. The longest, coding CDS transcript of each gene was used as input for wgd. The wgd pipeline acts as a wrapper for a number of programs, and in the case of the analysis here, the following programs were run through wgd: blastp (Altschul *et al*. 1997), MCL (Markov Cluster Process, Hazewinkel and Van Eijck 2000), PAML (Yang 2007), MAFFT (Katoh and Standley 2013), FastTree (Price *et al*. 2010), and i-ADHoRe 3.0 (Proost *et al*. 2012). In addition to wgd, OrthoFinder v2.5.4 (Emms and Kelly 2019) was run to discover orthologous groups unique to each species and shared between species. For OrthoFinder analyses, the longest peptide isoform for each gene was used as input. A full list of taxa included in OrthoFinder and doubletrouble analyses (described below) is given in Supplementary Table 3.

CAFE5 v5.1.0 (Mendes *et al*. 2021) was used to discover hierarchical orthogroups from OrthoFinder undergoing phylogenetically significant gene family expansions or contractions. To begin, r8s v1.81 (Sanderson 2003) was used to time-calibrate the phylogeny from OrthoFinder using fossil priors obtained from the PaleoBioDB fossil record (Peters and McClennen 2016). Priors for *Acropora palmata* (3.6 MYA, Budd *et al.* 1999), *Porites compressa* (2.588 MYA, Faichney *et al.* 2011), *Acropora* (59 MYA, Vecsei and Moussavian 1997), Faviina (247 MYA, Qi 1984), and Scleractinia (268 MYA, Gregorio 1930) were used as calibration points. With significantly expanding or contracting hierarchical orthogroups identified by CAFE5, GO terms for genes in expanding and contracting gene families were extracted and compared with the whole-genome background in each species to test for enrichment. Enrichment analyses were performed using GOAtools (Klopfenstein *et al*. 2018) on genes in expanding gene families not annotated as transposons and transposases. Genes functionally annotated with the transposition GO term (GO:0032196) and its child terms were also excluded. GO term enrichment was also assessed for orthogroups unique to each species (unshared orthogroups). To reduce false discovery, only terms with a Benjamini–Hochberg adjusted *P*-value <0.05, depth >2, and terms present in more than 5 study genes (all genes present in expanding orthogroups) were preserved. As GOAtools propagates child term counts up to parent terms, results can contain high redundancy and semantic similarity. To reduce some of the redundancy in significant GO terms, REVIGO v1.8.1 (Supek *et al*. 2011) was run using the SimRel semantic similarity measure to simplify enrichment results.

To classify stony coral (Scleractinia) paralogs into duplication types, doubletrouble v1.3.6 (Almeida-Silva and Van de Peer 2025) was run using the longest peptide isoform for each gene and default arguments. Briefly, doubletrouble classifies genes into segmental (SD), tandem (TD), proximal (PD), transposon-derived (TRD), and dispersed duplications (DD) based on collinearity, intron content, and phylogenetic position of paralogs. For instance, duplications are classified as tandem if 2 paralogs are separated by fewer than 10 genes. If the distance between genes is >10, paralogs are classified as PD. DD are considered any duplication that is not otherwise classifiable into more specific categories. For all doubletrouble analyses, *Amplexidiscus fenestrafer* (Wang *et al*. 2017), a member of the naked corals, Corallimorpharia, was used as an outgroup. Not all gene annotations were compatible with the “full” scheme, where TRD duplications are further classified into retrotransposon-derived and DNA transposon-derived. As such, the “full” scheme was only utilized for the focal study species here, *C. natans*, *D. cylindrus*, and *S. siderea*. All other species were run using the “extended” scheme.

## Results and discussion

### Assembly contiguity, completeness, and heterozygosity

All assemblies exhibit high contiguity (Table 1) and are gap-free. The *S. siderea* genome is roughly 2 times larger than observed in other corals species, with an assembly size of 822 M, compared with 526 and 399 Mb for *D. cylindrus* and *C. natans*, respectively. The assembly size of *S. siderea* is larger than most publicly available coral genome assemblies—only 2 species have larger assemblies, *Pachyseris speciosa* (Bongaerts *et al*. 2021) and *Platygyra sinensis* (Pootakham *et al*. 2021). However, the *P. sinensis* assembly likely contains considerable haplotig duplication, leaving only *P. speciosa* as a comparable assembly. In addition to being the largest of the 3 assemblies presented here, the *S. siderea* assembly is the most contiguous assembly (N50 = 9.1 Mb), likely due to the larger read N50 of SMRTcell 3 (see Supplementary Table 1). The genomes of *C. natans* and *D. cylindrus* have N50s of 4.647 and 4.902 Mb, respectively. Further scaffolding with Hi-C data could help elevate these 3 references to chromosome level. Genome-wide GC content is similar across all 3 species, with 39.81% for *S. siderea*, 38.87% for *C. natans*, and 39.29% for *D. cylindrus*. GC estimates are similar to other published stony coral genomes (e.g. Bongaerts *et al.* 2021).

**Table 1.** Assembly summary statistics for *Colpophyllia natans*, *Dendrogyra cylindrus*, and *Siderastrea siderea*.

	*Siderastrea siderea*	*Dendrogyra cylindrus*	*Colpophyllia natans*
Contig total (Mb)	822.514	526.444	398.943
Gap percentage	0%	0%	0%
Number of contigs	265	301	174
Contig N50	9.1 Mb	4.647 Mb	4.902 Mb
Largest contig	25.215 Mb	21.044 Mb	14.745 Mb
GC content (%)	39.81	39.29	38.87
% of k-mer estimate recovered	105.61	105.99	103.47
BUSCO Metazoa, complete (%)—genome mode	96.6	96.5	97.2
Single copy (genome)	93.9	95.3	96.1
Duplicated (genome)	2.7	1.3	1.0
Fragmented (%) (genome)	0.9	0.8	1.0
Missing (%) (genome)	2.4	2.6	1.8
BUSCO Metazoa, complete (%)—protein mode	91.8	94.9	95.5
Single copy (protein)	87.1	88.2	88.8
Duplicated (protein)	4.7	6.7	6.7
Fragmented (%) (protein)	3.2	1.8	2.0
Missing (%) (protein)	4.9	3.4	2.5
Gene models	61,712	39,739	34,139
Protein-coding gene models	52,473	34,738	29,090

**Fig. 1.**
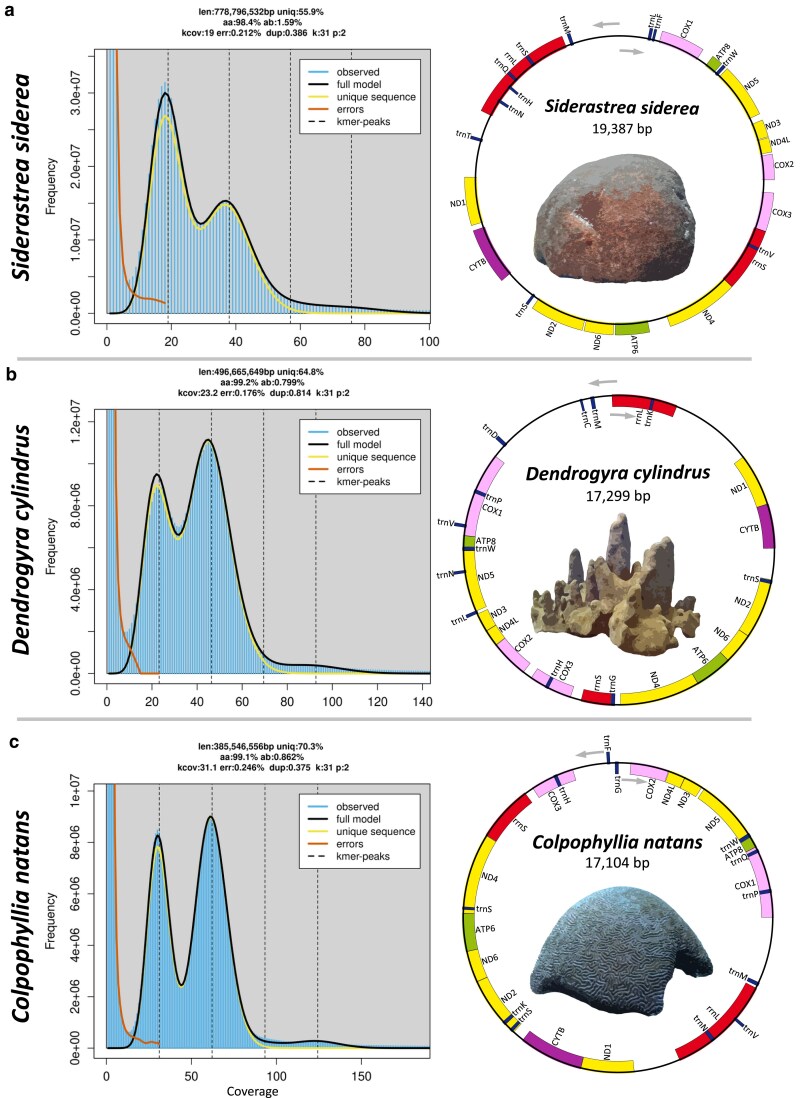
K-mer multiplicity plots (left panels) from GenomeScope2 (Ranallo-Benavidez *et al*. 2020) for a k-mer size of 31 for a) *Siderastrea siderea*, b) *Dendrogyra cylindrus*, and c) *Colpophyllia natans*. Mitochondrial genome gene order (right panels) in *S. siderea*, *D. cylindrus*, and *C. natans.* Mitogenomes assembled using MitoHiFi (Gabriel *et al*. 2023).

K-mer duplicity plots from GenomeScope2 (Fig. 1) suggest that all species here are diploid in nature, unlike the recent findings in Hawaiian corals (Stephens *et al*. 2022). All 3 assemblies exhibited high completeness as determined by BUSCO Metazoa in genome mode (Manni *et al*. 2021), with *C. natans*, *D. cylindrus*, and *S. siderea* showing 97.2, 96.5, and 96.6% completeness, respectively (Table 1). In terms of core BUSCO genes, *S. siderea* has the highest number of duplicated genes, with 2.7% of metazoan genes being duplicated. In genome mode, BUSCO showed that all 3 assemblies had similar percentages of fragmented and missing BUSCO genes. Evaluation of all protein isoforms using BUSCO in protein mode suggested that the complexity of the *S. siderea* genome may have slightly reduced the efficacy of genome annotation compared with the remaining 2 species, with completeness scores of 95.5, 94.9, and 91.8% for *C. natans*, *D. cylindrus*, and *S. siderea*, respectively. All assemblies are similar to their GenomeScope2 k-mer-based size estimates (Fig. 1 and Table 1). Taken together, these results suggest that the majority of all 3 genomes were successfully captured and annotated in our assemblies with little remaining haplotig duplication.

Genome-wide heterozygosity in corals typically ranges from 1.07 to 1.96% (Shinzato *et al*. 2021; Stephens *et al*. 2022; Yu *et al*. 2022; Young *et al*. 2024). K-mer frequency-based estimates of genome-wide heterozygosity from GenomeScope2 suggest that *D. cylindrus* has the lowest heterozygosity of the 3 species discussed here (0.799%) and among the lowest in any coral species for which genomic resources are available (Shinzato *et al*. 2021; Stephens *et al*. 2022; Yu *et al*. 2022; Young *et al*. 2024). The species has been rare throughout history (Hunter and Jones 1996; Modys *et al*. 2023) but with high local abundances in some locations (e.g. St. Thomas in the U.S. Virgin Islands). Recent catastrophic declines due to stony coral tissue loss disease (Neely *et al*. 2021; Alvarez-Filip *et al*. 2022) have led to the listing of the species as critically endangered by the International Union for Conservation of Nature (IUCN, Cavada-Blanco *et al.* 2022). *D. cylindrus* is extinct in the wild in Florida (Neely *et al*. 2021), and all genets are now in captivity. Captive-based spawning efforts are burgeoning (Craggs *et al*. 2017; O’Neil *et al*. 2021) to recover the species. The very low heterozygosity estimate provided here highlights the need for carefully managed breeding (Marhaver *et al*. 2015) to ensure the persistence of the remaining standing genetic variation and adaptive potential of *D. cylindrus* (Barrett and Schluter 2008; Kardos *et al*. 2021). Of the 3 species, *S. siderea* has the highest genome-wide heterozygosity estimate of 1.59% and *C. natans* is intermediate with 0.862%. *C. natans* also has low genome-wide heterozygosity compared with other coral species and may require genetic management in the future. However, these genome-wide heterozygosity estimates are generated from singular genets and may not accurately represent the heterozygosity of the wider populations of each species. *C. natans* is the only species discussed here that does not have range-wide population genetic information available. As such, further genetic characterization of the species is clearly warranted due to population declines caused by infectious diseases (Alvarez-Filip *et al*. 2022) and the heterozygosity estimates provided here.

### Repetitive content and transposable elements

The proportion of repeats assigned to each repeat category of RepeatMasker was similar across all 3 species assembled here (Table 2). Repetitive content was 47.80, 40.40, and 23.62% in *S. siderea*, *D. cylindrus*, and *C. natans*, respectively. The majority of repeats were interspersed, with unclassified repeats being most abundant in all 3 species (31.91, 25.57, and 12.22%). Compared with other cnidarians, these assemblies contain similar levels of repetitive content to jellyfish species such as members of *Clytia*, *Aurelia*, and *Chrysaora* containing 39–49.5% (Gold *et al*. 2019; Leclère *et al*. 2019; Xia *et al*. 2020) and to other scleractinian corals containing 13.6–58.1% (e.g. Shinzato *et al*. 2011; Cooke *et al*. 2020; Bongaerts *et al.* 2021; Kim *et al.* 2022; Stephens *et al.* 2022; Locatelli *et al*. 2024; Young *et al.* 2024). In our set of 3 species, we observe a general relationship of increasing repetitive content with increasing with genome size, corroborating that repeat expansion may be important in driving genome size disparities across evolutionary time in stony corals, as similarly observed in zoantharian and *Hydra* genomes (Wong *et al*. 2019; Fourreau *et al*. 2023).

**Table 2.** Repetitive content and transposable elements identified by RepeatMasker (Smit*et al*.) across *Siderastrea siderea*, *Dendrogyra cylindrus*, and *Colpophyllia natans*.

		*Siderastrea siderea*			*Dendrogyra cylindrus*			*Colpophyllia natans*		
DNA	Total	138,779	68,819,835	8.39%	82,872	47,945,358	9.09%	68,722	17,971,690	4.52%
	Maverick	22,353	42,786,635	5.20%	13,811	35,496,289	6.74%	5,091	5,626,678	1.41%
	Sola-3	17,315	6,171,939	0.75%	4,051	1,802,393	0.34%	3,249	1,019,211	0.26%
	PIF-Harbinger	9,247	1,246,387	0.15%	9,092	1,764,101	0.34%	5,361	821,524	0.21%
	Academ-1	4,662	1,873,178	0.23%	2,578	872,091	0.17%	1,886	777,963	0.20%
LINE	Total	104,185	32,929,736	4.02%	53,305	17,696,634	3.36%	46,166	15,626,622	3.92%
	Penelope	38,138	11,157,756	1.36%	16,584	5,217,438	0.99%	21,897	5,971,804	1.50%
	L1-Tx1	13,255	8,443,143	1.03%	9,745	5,273,753	1.00%	6,122	3,626,933	0.91%
	L2	29,218	6,994,614	0.85%	16,810	3,452,973	0.66%	11,440	3,468,236	0.87%
	RTE-BovB	4,796	946,003	0.12%	2,582	1,414,380	0.27%	2,717	1,144,012	0.29%
LTR	Total	36,888	17,315,718	2.09%	11,593	7,626,581	1.44%	10,698	7,645,820	1.92%
	Gypsy	14,784	5,919,375	0.72%	4,918	2,999,690	0.57%	5,407	4,098,188	1.03%
	Pao	4,227	4,683,913	0.57%	3,053	2,945,104	0.56%	1,809	1,501,851	0.38%
	DIRS	3,419	2,371,017	0.29%	1,323	882,015	0.17%	2,095	1,383,508	0.35%
	Ngaro	7,195	3,053,083	0.37%	869	469,818	0.09%	1,057	496,392	0.12%
SINE	Total	16,023	2,146,747	0.26%	4,485	541,212	0.10%	5,616	674,504	0.17%
	tRNA-V	3,912	567,988	0.07%	2,623	350,202	0.07%	3,166	512,682	0.13%
	MIR	8,147	1,173,564	0.14%	0	0	0.00%	0	0	0.00%
	tRNA-RTE	1,612	150,650	0.02%	1,010	100,831	0.02%	0	0	0.00%
	Alu	1,485	131,235	0.02%	0	0	0.00%	0	0	0.00%
Low complexity		455	76,303	0.01%	282	53,301	0.01%	123	26,366	0.01%
Retroposon	L1-dep	175	32,571	0.00%	0	0	0.00%	0	0	0.00%
Rolling circle	Helitron	2,701	1,113,967	0.14%	837	186,063	0.04%	5,529	2,007,025	0.50%
Satellites		476	226,250	0.03%	1,120	113,510	0.02%	1,795	189,861	0.05%
Simple repeats		17,276	2,670,182	0.32%	11,761	1,849,810	0.35%	6,765	1,167,479	0.29%
RNA repeats	Total	24,191	5,349,132	0.65%	21,009	2,054,088	0.39%	727	135,302	0.03%
	tRNA	23,498	5,198,251	0.63%	20,541	1,915,611	0.36%	395	44,379	0.01%
	rRNA	693	150,881	0.02%	468	138,477	0.03%	260	82,379	0.02%
	snRNA	0	0	0.00%	0	0	0.00%	72	8,544	0.00%
Unclassified		1,000,623	262,395,404	31.91%	635,176	134,635,802	25.57%	262,664	48,767,859	12.22%
Total		1,341,772	393,075,845	47.80%	822,440	212,702,359	40.40%	408,805	94,212,528	23.62%

The top 3 repeat families (e.g. Maverick) within each major repeat class (DNA, LINE, LTR, SINE, and RNA repeats) are presented in this table.

In *C. natans*, the DNA transposon class of repeats was reduced by ∼50% when compared with *S. siderea* and *D. cylindrus* (Table 2). Within the DNA transposons, the Maverick subclass represented the most prominent deficits in *C. natans*, representing only 1.41% of the genome compared with 5.20 and 6.74% in *S. siderea* and *D. cylindrus*, respectively. It is unclear whether DNA transposons have contracted in *C. natans* or expanded in *S. siderea* and *D. cylindrus*, although both purging and expansions of repetitive content have been implicated in genome size evolution in cnidarians and other organisms (Hawkins *et al*. 2006; Michael 2014; Roessler *et al*. 2019; Kon *et al*. 2024). Further work is required to understand the genomic processes by which repetitive DNA expands and contracts in cnidarian genomes, as well as the overall importance of repetitive content in speciation processes and establishing new lineages. However, the genome assemblies presented here echo the standing literature and suggest that losses or gains of certain repetitive classes exist across the diversity of extant stony corals.

### Gene prediction and unique orthogroups

*S. siderea* is unique among the assembled genomes not just for its size and contiguity, but also its gene content. Gene prediction in funannotate identified 61,712 gene models, roughly double the number of genes discovered for *D. cylindrus* and *C. natans* (39,739 and 34,139, respectively; Table 1), and compared with other publicly available coral genome assemblies (e.g. Prada *et al.* 2016; Fuller *et al.* 2020). Of these gene models, 52,473, 34,738, and 29,090 were predicted to be protein-coding for *S. siderea*, *D. cylindrus*, and *C. natans*, respectively. *D. cylindrus* and *C. natans* fall within the expectations for stony corals in terms of protein-coding gene content. The gene content of *S. siderea* is higher than expected, only comparable to *M. capitata* among published genomes (Stephens *et al*. 2022). Of the protein-coding gene models, 1,515, 297, and 287 models in *S. siderea*, *D. cylindrus*, and *C. natans* contained ≥90% repeat-masked bases, suggesting that these models may be derived from repetitive DNA and transposition-related events. A further 79, 47, and 31 gene models in *S. siderea*, *D. cylindrus*, and *C. natans* were either directly annotated as transposons or transposases or were associated with transposition (GO:0032196) or transposase activity (GO:0004803) related GO terms.

Because of the doubling in overall size and gene content present in the *S. siderea*, Ks tests were performed to test for an ancient whole-genome duplication in the evolution of the species. Ks distributions in species having experienced whole-genome duplication events exhibit characteristic distributions with a hump (as in Zwaenepoel and Van De Peer 2019), where many gene pairs are derived from a simultaneous duplication event and have all experienced a similar number of synonymous substitutions per synonymous site. Whole-genome duplication analyses in wgd did not find Ks ratios indicative of ancient whole-genome duplication in any of the species assembled here (Supplementary Fig. 1), suggesting that other processes may be responsible for gain in genome size. BUSCO completeness, as described above, also suggested that duplication of metazoan single copy genes in the *S. siderea* genome is minimal, further reducing support for a whole-genome duplication event.

**Fig. 2.**
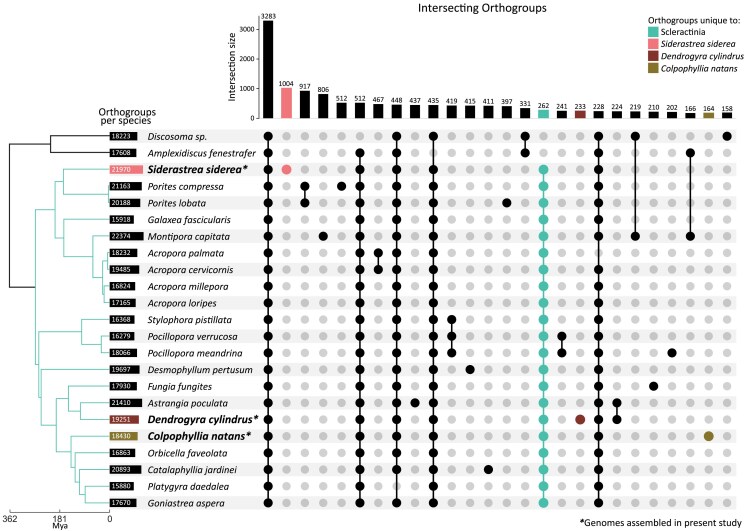
Upset plot describing unique and shared orthogroups across scleractinian corals and an outgroup, Corallimorpharia. Gene models were assigned to orthogroups using OrthoFinder (Emms and Kelly 2019). All included taxa are listed in Supplementary Table 3. The focal taxa assembled in the present study are indicated by bold font and asterisks (*).

OrthoFinder analyses placed 47,786 protein-coding genes into 21,970 orthogroups in *S. siderea*. Of these, 1,004 orthogroups containing 3,849 protein-coding genes were exclusively found in *S. siderea* (Fig. 2). An additional 4,687 protein-coding genes could not be binned into orthogroups by OrthoFinder. As OrthoFinder utilizes DIAMOND (Buchfink *et al*. 2015) with the --more-sensitive alignment option, orthogroups are only formed if inter- and intraspecies alignments are ≥40% in identity. The presence of thousands of unbinned genes and orthogroups unique to *S. siderea* suggests that gene duplication and subsequent diversification are prominent in the lineage. Among multicopy gene families unique to *S. siderea* (Supplementary Fig. 2), the most enriched GO term compared with the genomic background was “bioluminescence” (GO:0008218). Fluorescent pigment proteins have been shown to undergo rapid evolution and strong selection in corals (Voolstra *et al*. 2011). These proteins are photoprotective for the coral holobiont (Salih *et al*. 2000) and can serve to optimize the light environment of symbiotic Symbiodiniaceae (Bollati *et al*. 2022). Indeed, presence of pink fluorescent pigment in congener *Siderastrea stellata* is associated with higher temperatures (Tunala *et al*. 2023). *S. siderea* harbors 3 distinct genetic lineages (Aichelman *et al*. 2025) of which only one was sequenced here. Additional genome assemblies of the other 2 lineages may shed light on the taxonomic status of these lineages and what role gene duplication and diversification may have played in their evolution.

*D. cylindrus* had comparatively fewer protein-coding genes placed into a similar number of orthogroups—32,896 genes in 19,251 orthogroups. Of these, 233 orthogroups were unique to the species, containing a total of 1,061 genes. 1,842 genes remained unbinned. Of the orthogroups unique to *D. cylindrus*, the most enriched GO term was “response to defense of other organism” (GO:0098542, Supplementary Fig. 2). The lack of specificity of the term makes it unclear whether this refers to external organisms (e.g. damage from predation or disease) or internal organisms (e.g. intracellular Symbiodiniaceae symbionts). However, a child term of GO:0098542, “defense response to bacterium” (GO:0042742), is found among the genes in gene families unique to *D. cylindrus*, suggesting that immune response to infection is particularly important to the species. Other enriched GO terms such as “apoptotic process” and “regulation of response to external stimulus” further support that response to bacterial infection may be particularly important to the species. Given its lineage age, *D. cylindrus* has been suggested to be intrinsically better at fighting infections compared with younger lineages (Pinzón *et al*. 2014). Recent catastrophic losses of the species due to stony coral tissue loss disease may have broken this long-standing advantage (Alvarez-Filip *et al*. 2022), although some evidence suggests that the disease may be the result of an infection of the symbiont that cascades to affect the host (Klein *et al*. 2024), rather than directly infecting the host.

*C. natans* had the fewest orthogroups, with 27,892 protein-coding genes placed into 18,430 orthogroups. 545 genes were placed into 164 orthogroups that were unique to the species and 1,198 genes remained unbinned. There was an enrichment for terms relating to transfer RNA (tRNA) modification (GO:0002949 and GO:0070525) in the gene families only found in *C. natans* (Supplementary Fig. 2). “Bioluminescence” also appears among the most enriched GO terms in orthogroups unique to the species, similar to *S. siderea*. Additionally, several terms relating to growth and development (“blastocyst growth” and “anatomical structure maturation”) were found to be enriched among orthogroups unique to *C. natans*. *C. natans* is among the most quickly developing broadcast spawners in the Caribbean, with settlement and the onset of zooplanktivory occurring in as little as 3–4 days (Geertsma *et al*. 2022; Yus *et al*. 2024), possibly due to the enrichment of growth-related terms observed here. The “regulation of pH” is also enriched, perhaps allowing the species to survive in environments less conducive to survival in other species. For instance, *C. natans* is one of the few coral species able to thrive in unusual habitats such as mangrove canopy environments with comparatively low pH, as well as reef flats and reef slope environments more typically associated with Caribbean reef communities (Stewart *et al*. 2022).

### Mitochondrial genomes

Mitochondrial genomes were successfully assembled for all 3 species discussed here using MitoHiFi (Gabriel *et al*. 2023). Both *D. cylindrus* and *C. natans* were of similar size with lengths of 17,299 and 17,104 bp, respectively. *S. siderea* is considerably larger, with a total length of 19,387 bp (Fig. 1). The *S. siderea* mitogenome is among the largest of all stony coral (Scleractinia). Of all sequenced scleractinians, the mitogenome of *S. siderea* is exceeded in length only by the solitary coral species *Polymyces wellsi* (Flabellidae, NC_082103.1, 19,924 bp), *Deltocyathus magnificus* (Deltocyathidae, OR625187.1, 19,736 bp), and *Rhombopsammia niphada* (Micrabaciidae, MT706034.1, 19,654 bp), and colony-forming species *Pseudosiderastrea formosa* and *Pseudosiderastrea tayami* (Siderastreidae, NC_026530.1 and NC_026531.1, 19,475 bp). In terms of gene structure, all 3 mitochondrial genome assemblies consist of 13 protein-coding genes and 2 ribosomal RNA (rRNA, rrnL and rrnS) genes with highly conserved gene order (ND5, ATP8, COX1, rrnL, ND1, CYTB, ND2, ND6, ATP6, ND4, rrnS, COX3, COX2, ND4L, and ND3). Both *D. cylindrus* and *C. natans* contain 12 tRNA genes, while *S. siderea* contains 11.

### Expansion of shared gene families and modes of duplication

**Fig. 3.**
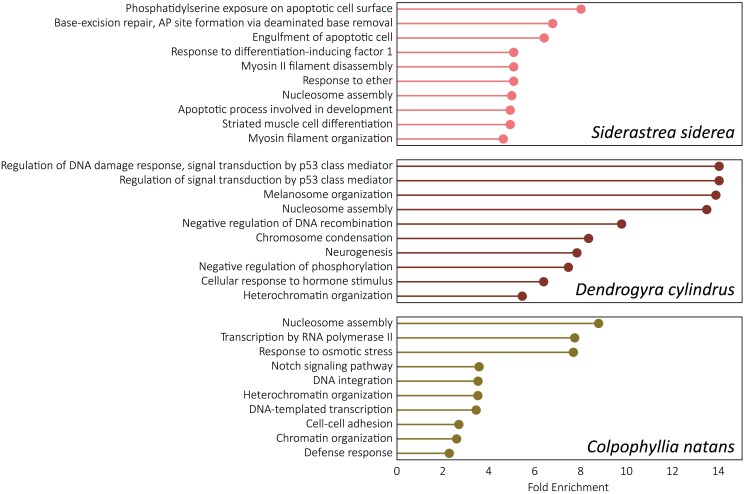
Top 10 GO terms enriched in orthogroups undergoing phylogenetically significant expansion in *Siderastrea siderea*, *Dendrogyra cylindrus*, and *Colpophyllia natans*. Orthogroups were assigned using OrthoFinder (Emms and Kelly 2019). Gene families undergoing phylogenetically significant expansion were identified using CAFE5 (Mendes *et al*. 2021). GO enrichment analyses were performed in GOATools (Klopfenstein *et al*. 2018).

GO enrichment analyses of shared gene families undergoing phylogenetically significant expansion (as identified by OrthoFinder and CAFE5) may point to the importance of specific functional attributes in the evolution of each of the taxa assembled here (Fig. 3). In all 3 species, there was an enrichment among significantly expanding gene families for GO terms relating to nucleosome assembly, chromosome condensation, and chromatin/heterochromatin organization when compared with the genomic background of each species. This suggests that these functional attributes were disproportionately important in the evolution of stony corals. Chromatin accessibility is important for fine-tuning transcriptional response (GO:0006351 and GO:0006366, enriched in *C. natans*), as well as DNA repair (GO:0097510, enriched in *S. siderea*) and recombination (GO:0045910 and GO:0015074, enriched in *D. cylindrus* and *C. natans*, respectively) (Tsompana and Buck 2014). Experiments in the model sea anemones, *Nematostella* and *Aiptasia*, further corroborate this hypothesis, demonstrating that chromatin accessibility is dynamic over the course of stressful events such as heat exposure and resulted in expressional changes in pathways related to immune response, oxidative stress, metabolism, and DNA repair (Weizman and Levy 2019; Weizman *et al*. 2021).

In *S. siderea*, “phosphatidylserine exposure on apoptotic cell surface” (GO:0070782) is the most enriched GO term in gene families that are significantly expanding compared with the genomic background of the species (Fig. 3). Additionally, the terms “engulfment of apoptotic cell” (GO:0043652) and “apoptotic process involved in development” (GO:1902742) were also enriched in *S. siderea*. Stress response in corals involves the activation of apoptotic pathways, particularly in the case of heat stress response (Kvitt *et al*. 2011; Tchernov *et al*. 2011; Helgoe *et al*. 2024), and *S. siderea* is among the most heat-tolerant corals inhabiting Caribbean reefs (Palacio-Castro *et al*. 2021). The expansion of apoptosis-regulating gene families may be, in part, responsible for the overall resilience of *S. siderea* to adverse environmental conditions. In addition to apoptotic pathways and chromatin structure, there was also an enrichment of multiple myosin- and muscle-related ontology terms (GO:0031035, GO:0031033, GO:0051146). Myosin proteins have been identified as differentially expressed or differentially concentrated across inshore-offshore gradients, in heat stress conditions, and in diseased tissue across divergent coral taxa and may also play a role in *S. siderea*'s resilience (DeSalvo *et al*. 2010; Ricaurte *et al*. 2016; Wong *et al*. 2021; Mayfield 2022).

The 2 GO terms with the highest fold enrichment in *D. cylindrus* both involve pathways of the p53 class mediator (GO:1901796 and GO:0043516), one of which involves the regulation of DNA damage response. Sessile, shallow-living marine organisms are exposed to high levels of UV radiation, and corals have fast and effective DNA repair mechanisms at all life stages (Reef *et al*. 2009; Svanfeldt *et al*. 2014). Melanosome organization (GO:0032438) was also found highly enriched in *D. cylindrus*. Melanin production is important in cnidarian innate immunity (Palmer *et al*. 2012; Changsut *et al*. 2022; Van Buren *et al*. 2024) and may serve to protect shallow-living corals from UV exposure (Wall *et al*. 2018), and also protect their symbionts (Harman *et al*. 2022). *D. cylindrus* is a long-lived species, and even colonies in early development with no vertical pillar formation may be older than 30 years (Neely *et al*. 2021). This longevity may explain the enrichment in processes that reduce UV exposure and repair DNA damage that accumulates during the life of a genet.

Compared with other species in the analysis, gene families most expanded in *C. natans* were enriched in functions related to environmental response (“response to osmotic stress,” GO:0006970) as well as cell signaling (“Notch signaling pathway,” GO:0007219 and “cell–cell adhesion,” GO:0098609) and immune response (“defense response,” GO:0006952). As described above, *C. natans* is able to persist in habitats such as mangrove stilt roots. These environments are often low in pH and low in salinity, and expansions of gene families relating to osmotic stress response may enable the species to thrive in these challenging habitats. The remainder of the expanded gene families in *C. natans* were involved in chromatin accessibility and nucleosome assembly (Fig. 3) as in *S. siderea* and *D. cylindrus*.

Subsequent analysis of paralogs using doubletrouble found that proximal duplications (PD—locally duplicated with paralogs separated by 10 or more genes) were the most prominent form of classifiable gene duplications in *S. siderea* (Supplementary Fig. 3 and Supplementary Table 4). Previous studies have suggested that tandem duplications (TD) drive Scleractinian (stony coral) evolution (Noel *et al*. 2023). Indeed, TD appeared to be more abundant in *S. siderea* in comparison with many of the evaluated taxa (Supplementary Fig. 3 and Supplementary Table 4). However, duplicate classification is inherently challenging as the order of genes can be the result of many different potential processes. For instance, TD can be broken apart by dispersed duplications (DD) being copied between tandem paralogs. These would resemble PD according to doubletrouble's classification schema, despite being the result of 2 separate duplication processes. Additionally, analyses comparing species are somewhat reliant on similarly high-quality annotation and assembly across analyzed taxa. Several of the assemblies evaluated in our duplication analyses are of low contiguity and filled with short-read derived gaps, which could reduce the ability to detect certain forms of duplication. For example, *O. faveolata* (Prada *et al.* 2016) contains no segmental duplications (SD, Supplementary Table 4), potentially because the detection of collinear, duplicated blocks of genes is less likely when the genome is highly fragmented. Further, it may not be possible to assign duplicates as transposon-derived duplications (TRD) with assemblies derived from Nanopore or PacBio CLR data (e.g. *Acropora cervicornis*, Locatelli *et al*. 2024). Even polished long-read assemblies may contain enough error in repetitive proteins such that a single copy of the gene cannot be assigned as ancestral—a requirement for paralogs to be classified as TRDs.

Despite the expansion of duplicated genes in Scleractinian species with larger genome sizes (e.g. *S. siderea* and *M. capitata*, Supplementary Fig. 3), tandemly duplicated genes do not appear to have a disproportionate impact on genome size or gene content as suggested previously (Noel *et al*. 2023). When all duplicates are scaled to a value of 1 (Supplementary Fig. 4), no singular duplication category appears to be most important in governing coral genome size. Instead, the proportion of paralogs assigned to each duplication type is similar across all species (an average of 22.0% tandem, 14.5% proximal, 2.3% segmental, 19.0% transposon-related, and 42.2% dispersed, Supplementary Table 4). This suggests that all duplication types are expanding in synchrony to result in the genome size disparities we see across the phylogeny of Scleractinia. Further expansion of duplication analyses to include assemblies from upcoming efforts of large database projects (e.g. Reef Genomics, Liew *et al.* 2016; Aquatic Symbiosis Genomics Project, McKenna *et al.* 2021) could help elucidate more fine-scale, lineage-specific duplication processes that we have been unable to capture here.

### Symbiont contigs

As metagenome assemblers were utilized in the assembly of the host species, symbiont data were also co-assembled and were of sufficient coverage to identify the prominent symbiont present to at least the genus-level. Both *C. natans* and *D. cylindrus* contained *Breviolum*, with *D. cylindrus* most likely containing *Breviolum dendrogyrum*, as described in Lewis *et al*. (2019). However, the top ITS2 hits (determined by *e*-value, followed by percent identity) for both species do not closely match formally named strains/species in the curated ITS2 database (*C. natans* top symbiont hit B4, 89.89%, *e*-value 3.33*e*−24; *D. cylindrus* top hit B1, 97.98%, *e*-value 2.21*e*−42). It is possible that the symbionts contained in the genome assembly samples of *C. natans* and *D. cylindrus* are not yet represented in this database.

In the initial separation of host and symbiont contigs using BlobTools, the *S. siderea* genet assembled here was found to be associated with *Cladocopium*, but comparison of contigs with the ITS2 database did not reveal any more specific hits. The psbA region is a more reliable marker for symbiont strain identification than ITS2 (LaJeunesse and Thornhill 2011). However, symbiont reference sequences for psbA are not currently as extensive as ITS2 in strain coverage. As the ITS2 and psbA databases continue to grow, symbiont contigs assembled here could be identified with greater taxonomic resolution.

In addition to eukaryotic algal symbionts, one notable prokaryotic symbiont was recovered. Within the assembly for *C. natans*, a 2.13-Mb contig was identified as most closely related to *Prosthecochloris aestuarii*. This bacterium has been proposed as a putatively symbiotic microbe living within coral skeletons (Cai *et al*. 2017; Chen *et al*. 2021). Coral metagenomes contain a wealth of symbionts with important functions for the holobiont (Bourne *et al*. 2009; Thompson *et al*. 2015; Boilard *et al*. 2020; Garrido *et al*. 2021). Further exploration of coral associated microbial communities may identify novel associations that are critical for the survival of the coral host.

### Conclusion

Here, we generated novel genome assemblies for key Caribbean reef-building corals, all of which are listed as vulnerable or critically endangered by the IUCN. All genome assemblies are highly complete (>95% BUSCO Metazoa) and contiguous (N50 > 4.6 Mb). The genomes of *D. cylindrus* and *C. natans* fall within nominal expectations of size and gene content based on other published coral genomes. *S. siderea* is roughly 2 times larger than expected with twice the number of predicted gene models, despite no evidence for a whole-genome duplication event. Repeat and gene family expansions seem to be drivers of the larger *S. siderea* genome size. These results align with and expand upon previously published literature which implicated gene duplications as a driving factor of stony coral evolution (Noel *et al*. 2023). Given the importance of duplications in speciation across corals, further work should explore intraspecific structural polymorphisms (such as copy number variants) to understand how structural variation plays a role in structure and adaptation at the population level.

These assemblies will help aid the broader research community by enabling high-resolution genomic analyses that explore trait variation within species and potentially provide restoration practitioners with useful information to implement in restoration initiatives. As coral populations continue their decline, it is crucial that we develop a thorough understanding of the genomic processes that have driven coral evolution and have allowed them to overcome past extinction events and global stressors. These reference assemblies provide a key stepping stone toward this goal.

## Supplementary Material

jkaf020_Supplementary_Data

## Data Availability

Raw sequencing data and assemblies generated for this project are available on the NCBI Sequence Read Archive (SRA) under BioProject accession PRJNA982825. These Whole Genome Shotgun projects (assemblies) have been deposited at DDBJ/ENA/GenBank under the accessions GCA_043250745.1, GCA_043250805.1, and GCA_043250775.1 for *Dendrogyra cylindrus*, *Colpophyllia natans*, and *Siderastrea siderea*, respectively. All annotations and associated assembly and analysis scripts and files are publicly available on Zenodo at https://zenodo.org/doi/10.5281/zenodo.13323697. Supplemental material available at G3 online.
