# Supplementary material for: Genomes of the Caribbean reef-building corals Colpophyllia natans, Dendrogyra cylindrus, and Siderastrea siderea
Source: G3 (Bethesda). 2025 Feb 1;15(4):jkaf020. doi: 10.1093/g3journal/jkaf020 (PMC12005156; doi:10.1093/g3journal/jkaf020)
Supplement: jkaf020_Supplementary_Data [file jkaf020_supplementary_data.zip › Supplemental_Captions.pdf]

Supplementary Data Captions and References

Nicolas S Locatelli, Iliana B Baums, Genomes of the Caribbean reef-building corals Colpophyllia natans, Dendrogyra cylindrus, and Siderastrea siderea, *G3 Genes|Genomes|Genetics*, 2025;, jkaf020, https://doi.org/10.1093/g3journal/jkaf020

Supplemental Tables

Table S1: Summary statistics of each Pacific BioSciences SMRTCell, for *Colpophyllia natans, Dendrogyra cylindrus*, and *Siderastrea siderea*. Gb=gigabases

Table S2: RNAseq accessions used for gene prediction of *Colpophyllia natans* and *Siderastrea siderea* genome assemblies.

Table S3: Genome assemblies used for OrthoFinder (Emms and Kelly 2019) and doubletrouble (Almeida-Silva and Van de Peer 2025) comparative analyses.

Table S4: All duplicate classifications identified by doubletrouble (Almeida-Silva and Van de Peer 2025). The "full" classification schema of doubletrouble was only run in *Colpophyllia natans, Dendrogyra cylindrus*, and *Siderastrea siderea* due to compatibility of input files. SD=Segmental duplicates, TD=Tandem duplicates, PD=Proximal duplicates, TRD=Transposon-derived duplicates, rTRD=Retrotransposon-derived duplicates, dTRD=DNA transposon-derived duplicates, and DD=Dispersed duplicates. Species in bold were assembled and annotated in this study. All included taxa are listed in **Table S3**.

Supplemental Figures

Fig. S1: Ks distribution plots generated by the wgd pipeline (Zwaenepoel and Van De Peer 2019). Ks plots were generated using the longest CDS transcript for each gene in each species. A secondary hump in the Ks distributions would support the presence of a whole genome duplication event. None of the three species here possess distributions that characterize whole genome duplications.

Fig. S2: Top 10 gene ontology (GO) terms enriched in orthogroups unique to *Siderastrea siderea, Dendrogyra cylindrus,* and *Colpophyllia natans*. Orthogroups were assigned using OrthoFinder (Emms and Kelly 2019). GO enrichment analyses were performed in GOATools (Klopfenstein et al. 2018).

Fig. S3: Gene duplication classes as identified by doubletrouble. Gene duplications were assigned duplication classes by doubletrouble (Almeida-Silva and Van de Peer 2025). Gene duplication is closely related to genome size, which is depicted to the left of species names. SD=Segmental duplication, TD=Tandem duplication, PD=Proximal duplication, TRD=Transposon-derived duplication, and DD=Dispersed duplication. The focal taxa assembled in the present study are indicated by bold font and asterisks (*). All included taxa are listed in **Table S3**.

Fig. S4: The proportion of paralogs assigned to each duplication category by doubletrouble (Almeida-Silva and Van de Peer 2025). SD=Segmental duplication, TD=Tandem duplication, PD=Proximal duplication, TRD=Transposon-related duplication, DD=Dispersed duplication. All included taxa are listed in **Table S3**.

References

Almeida-Silva F, Van de Peer Y. 2025. doubletrouble: an R/Bioconductor package for the identification, classification, and analysis of gene and genome duplications. Bioinformatics.:btaf043. doi:10.1093/bioinformatics/btaf043.

Buitrago-López C, Mariappan KG, Cárdenas A, Gegner HM, Voolstra CR. 2020. The Genome of the Cauliflower Coral Pocillopora verrucosa. Genome Biology and Evolution. 12(10):1911–1917. doi:10.1093/gbe/evaa184.

Emms DM, Kelly S. 2019. OrthoFinder: Phylogenetic orthology inference for comparative genomics. Genome Biology. 20(1):1–14. doi:10.1186/s13059-019-1832-y.

Fuller ZL, Mocellin VJL, Morris LA, Cantin N, Shepherd J, Sarre L, Peng J, Liao Y, Pickrell J, Andolfatto P, et al. 2020. Population genetics of the coral Acropora millepora: Toward genomic prediction of bleaching. Science. 369(6501). doi:10.1126/SCIENCE.ABA4674.

Helmkampf M, Bellinger MR, Geib SM, Sim SB, Takabayashi M. 2019. Draft Genome of the Rice Coral Montipora capitata Obtained from Linked-Read Sequencing. Genome Biology and Evolution. 11(7):2045–2054. doi:10.1093/gbe/evz135.

Herrera S, Cordes EE. 2023. Genome assembly of the deep-sea coral Lophelia pertusa. Gigabyte. 2023:1–12. doi:10.46471/gigabyte.78.

Klopfenstein DV, Zhang L, Pedersen BS, Ramírez F, Warwick Vesztrocy A, Naldi A, Mungall CJ, Yunes JM, Botvinnik O, Weigel M, et al. 2018. GOATOOLS: A Python library for Gene Ontology analyses. Sci Rep. 8(1):10872. doi:10.1038/s41598-018-28948-z.

Liew YJ, Aranda M, Voolstra CR. 2016. Reefgenomics.Org - a repository for marine genomics data. Database.

Locatelli NS, Kitchen SA, Stankiewicz KH, Osborne CC, Dellaert Z, Elder H, Kamel B, Koch HR, Fogarty ND, Baums IB. 2024. Chromosome-level genome assemblies and genetic maps reveal heterochiasmy and macrosynteny in endangered Atlantic Acropora. BMC Genomics. 25(1):1119. doi:10.1186/s12864-024-11025-3.

Noel B, Denoeud F, Rouan A, Buitrago-López C, Capasso L, Poulain J, Boissin E, Pousse M, Da Silva C, Couloux A, et al. 2023. Pervasive tandem duplications and convergent evolution shape coral genomes. Genome Biology. 24(1):123. doi:10.1186/s13059-023-02960-7.

Prada C, Hanna B, Budd AF, Woodley CM, Schmutz J, Grimwood J, Iglesias-Prieto R, Pandolfi JM, Levitan D, Johnson KG, et al. 2016. Empty Niches after Extinctions Increase Population Sizes of Modern Corals. Current Biology. 26(23):3190–3194. doi:10.1016/j.cub.2016.09.039.

Salazar OR, Prasanna N A, Cui G, Bay LK, van Oppen MJH, Webster NS, Aranda M. 2022. The coral Acropora loripes genome reveals an alternative pathway for cysteine biosynthesis in animals. Science Advances. 8(38):304. doi:10.1126/sciadv.abq0304.

Stankiewicz KH, Guiglielmoni N, Kitchen SA, Flot J-F, Barott KL, Davies SW, Finnerty JR, Grace SP, Kaufman LS, Putnam HM, et al. 2023. Genomic comparison of the temperate coral

Astrangia poculata with tropical corals yields insights into winter quiescence, innate immunity, and sexual reproduction. :2023.09.22.558704. doi:10.1101/2023.09.22.558704.

Stephens TG, Lee J, Jeong Y, Yoon HS, Putnam HM, Majerová E, Bhattacharya D. 2022. High-quality genome assemblies from key Hawaiian coral species. GigaScience. 11. doi:10.1093/gigascience/giac098.

Voolstra CR, Li Y, Liew YJ, Baumgarten S, Zoccola D, Flot J-F, Tambutté S, Allemand D, Aranda M. 2017. Comparative analysis of the genomes of Stylophora pistillata and Acropora digitifera provides evidence for extensive differences between species of corals. Sci Rep. 7(1):17583. doi:10.1038/s41598-017-17484-x.

Wang X, Liew YJ, Li Y, Zoccola D, Tambutte S, Aranda M. 2017. Draft genomes of the corallimorpharians Amplexidiscus fenestrafer and Discosoma sp. Molecular Ecology Resources. 17(6):e187–e195. doi:10.1111/1755-0998.12680.

Ying H, Cooke I, Sprungala S, Wang W, Hayward DC, Tang Y, Huttley G, Ball EE, Forêt S, Miller DJ. 2018. Comparative genomics reveals the distinct evolutionary trajectories of the robust and complex coral lineages. Genome Biology. 19(1):175. doi:10.1186/s13059-018-1552-8.

Yu Y, Nong W, So WL, Xie Y, Yip HY, Haimovitz J, Swale T, Baker DM, Bendena WG, Chan TF, et al. 2022. Genome of elegance coral Catalaphyllia jardinei (Euphylliidae). Frontiers in Marine Science. 9. doi:10.3389/fmars.2022.991391.

Zwaenepoel A, Van De Peer Y. 2019. Wgd-simple command line tools for the analysis of ancient whole-genome duplications. Bioinformatics. 35(12):2153–2155. doi:10.1093/bioinformatics/bty915.
