# Supplementary figures and images for: Genomes of the Caribbean reef-building corals Colpophyllia natans, Dendrogyra cylindrus, and Siderastrea siderea
Source: G3 (Bethesda). 2025 Feb 1;15(4):jkaf020. doi: 10.1093/g3journal/jkaf020 (PMC12005156; doi:10.1093/g3journal/jkaf020)

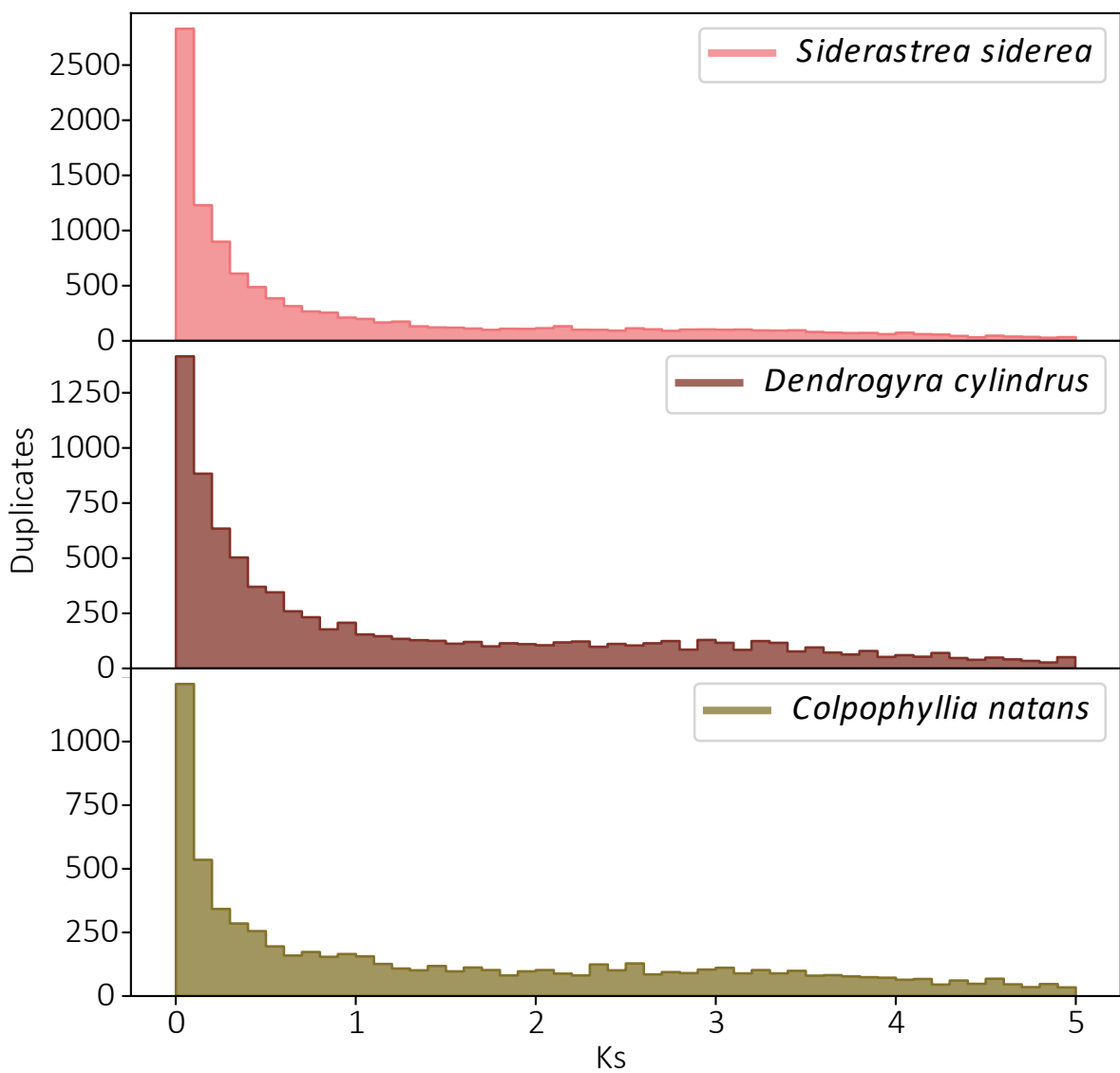

Supplement: jkaf020_Supplementary_Data [file jkaf020_supplementary_data.zip › Figure_S1_G3-2024-405555.pdf]

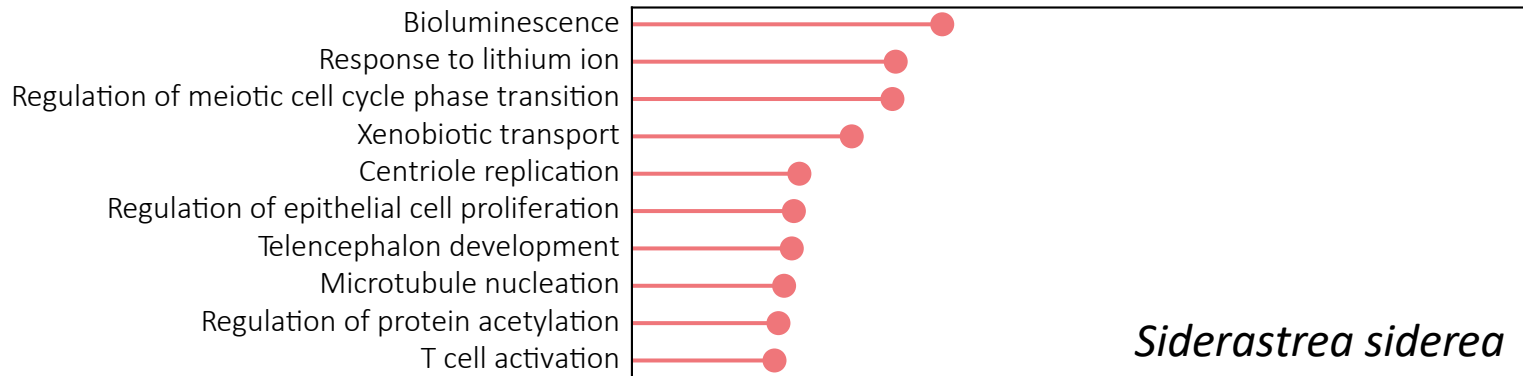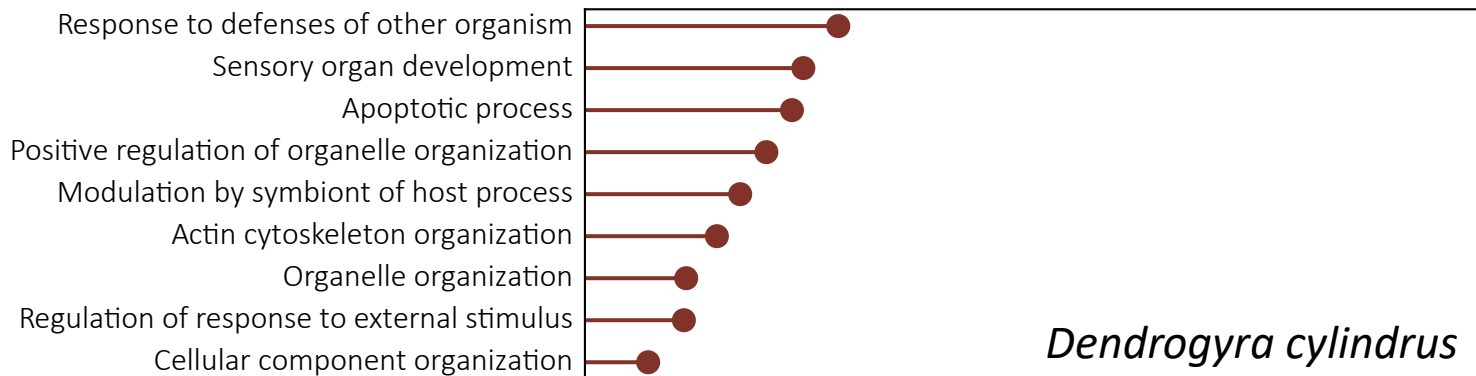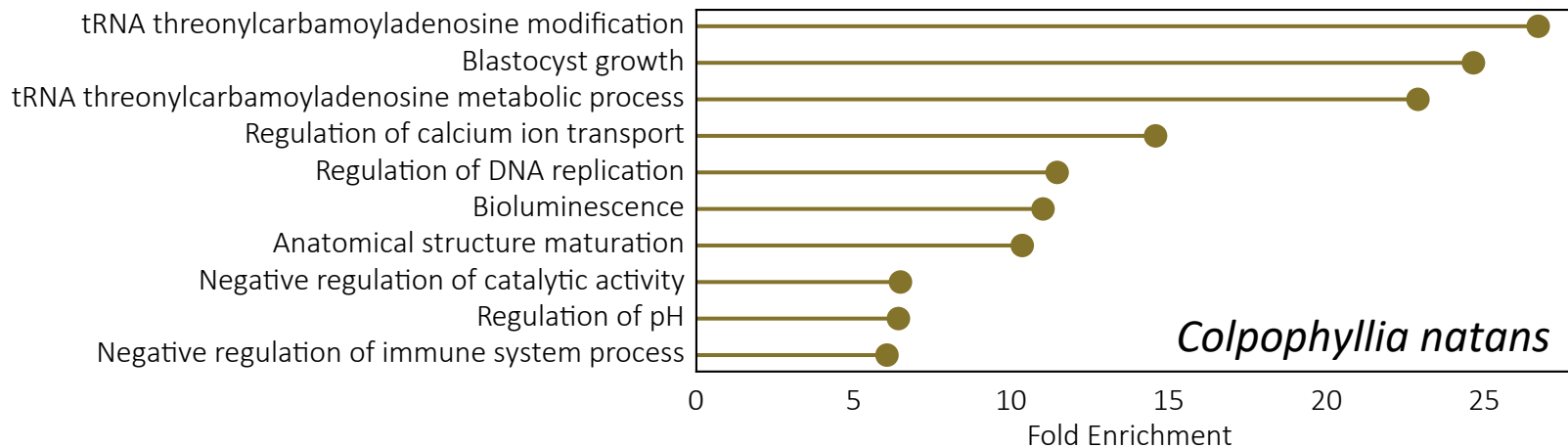

Supplement: jkaf020_Supplementary_Data [file jkaf020_supplementary_data.zip › Figure_S2_G3-2024-405555.pdf]

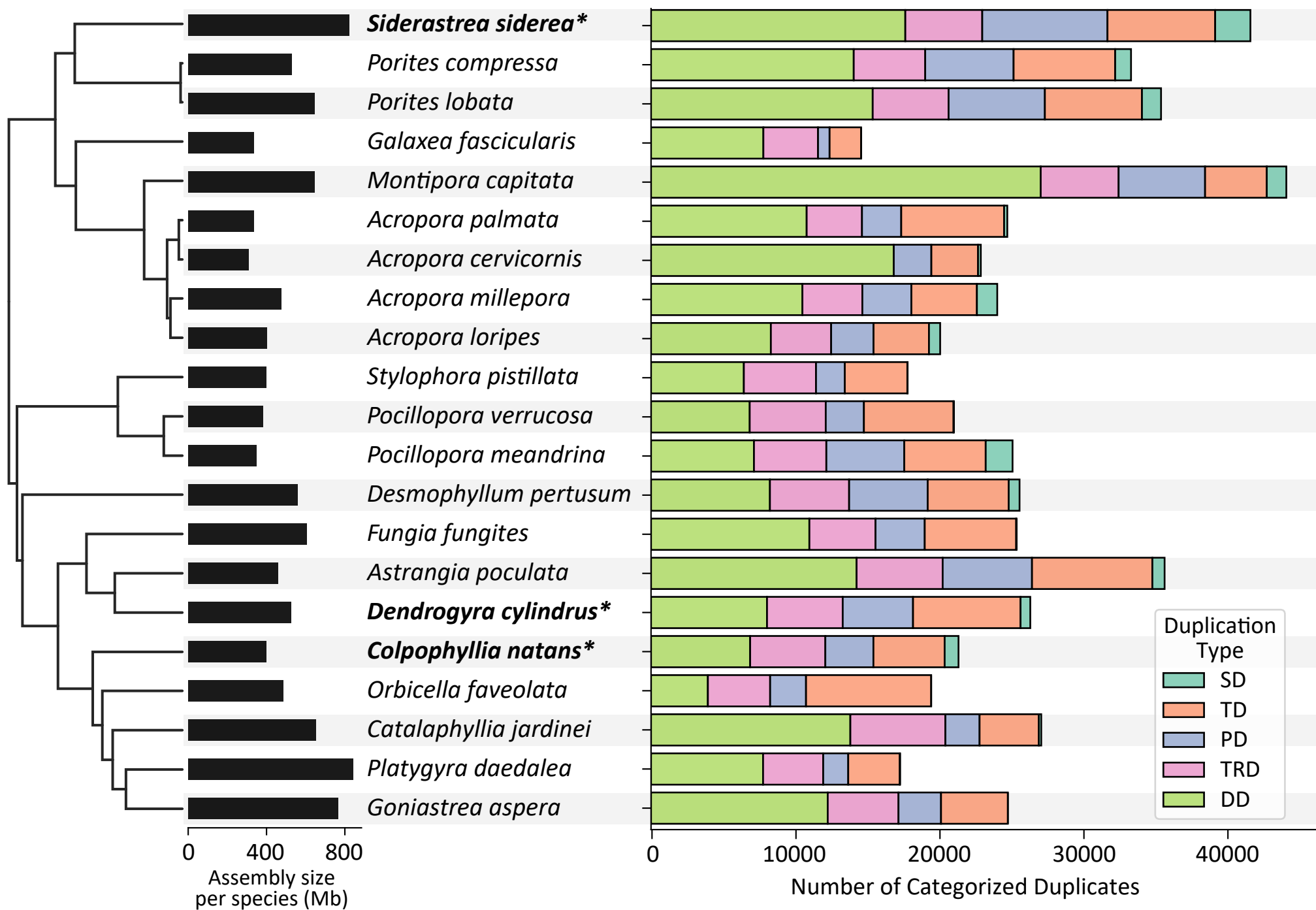

\*Genomes assembled in present study

Supplement: jkaf020_Supplementary_Data [file jkaf020_supplementary_data.zip › Figure_S3_G3-2024-405555.pdf]

Proportion of Categorized Duplicates

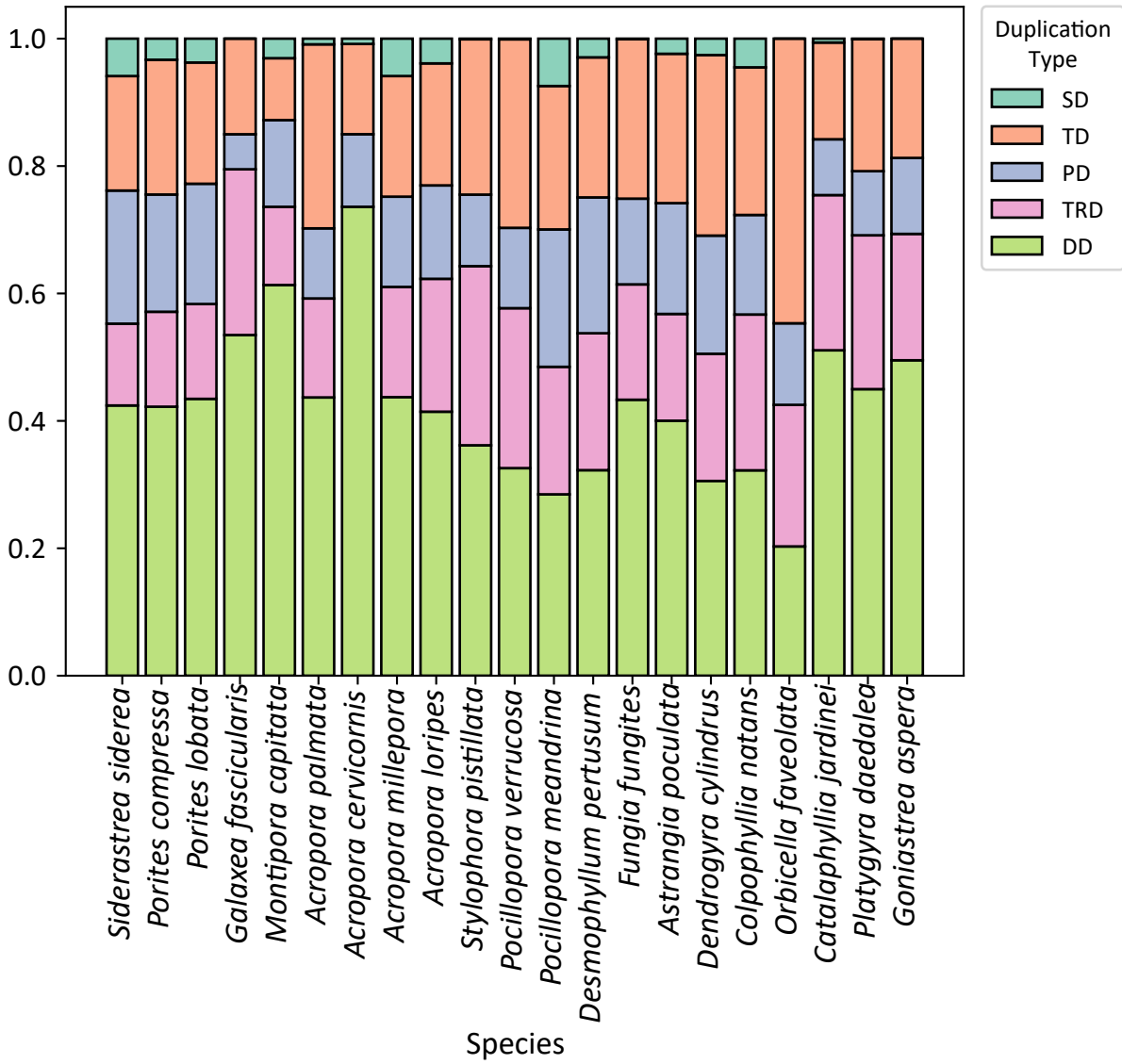

Supplement: jkaf020_Supplementary_Data [file jkaf020_supplementary_data.zip › Figure_S4_G3-2024-405555.pdf]
